# Atomic-resolution STEM-EDS studies of cation ordering in Ti-Nb oxide crystals

**DOI:** 10.1038/s41598-021-97244-0

**Published:** 2021-09-09

**Authors:** Sumio Iijima, Ichiro Ohnishi, Zheng Liu

**Affiliations:** 1 Meijo University, Graduate School of Science and Technology, Nagoya, 468-8502 Japan; 2 JEOL Ltd., 3-1-2 Musashino, Akishima, Tokyo 196-8558 Japan; 3 Innovative Functional Materials Research Institute, National Institute of Advanced Industrial Science and Technology (AIST), Nagoya, 463-8560 Japan

**Keywords:** Materials science, Physics

## Abstract

Ternary metal oxide compounds, such as Ti-Nb and Nb-W oxides, have renewed research interest in energy storage materials because these oxides contain multivalent metal ions that may be able to control the ion transport in solid lithium batteries. One of these oxides is Ti_2_Nb_10_O_29_, which is composed of metal–oxygen octahedra connected through corner-sharing and edge-sharing to form “block structures”. In the early 1970s Von Dreele and Cheetham proposed a metal-atoms ordering in this oxide crystal using Rietveld refined neutron powder diffraction method. Most recent studies on these oxides, however, have not considered cation ordering in evaluating the battery electrode materials. In this paper, by utilizing the latest scanning transmission electron microscopy combined with energy dispersive X-ray spectroscopy imaging technology, the cation chemical ordering in those oxide crystals was directly revealed at atomic resolution.

## Introduction

It is important to know the real crystal structures of any material in order to understand their physical and chemical properties as well as their practical uses. Crystal structures were traditionally analyzed by X-ray or neutron diffraction methods; however, these techniques are not adequate for cases where samples are powder crystals, nanocrystals (commonly used in Li-battery electrodes), or contain crystalline imperfections^1–8^. Nevertheless, these crystals can be studied with high-resolution transmission electron microscopy (HRTEM), and the usefulness of this method has been proved over the years^9^.

Ti-Nb-W powder oxide crystals are complex metal oxides that have been studied extensively by one of the present authors in the 1970s^10–18^. Many of these oxides showed nonstoichiometric compositions, which might be associated with some types of crystalline disorders and alter the electronic properties. Therefore, elucidation of the cation ordering of these ternary oxides is of great interest in energy storage material research. Wadsley first reported the crystal structure of Ti_2_Nb_10_O_29_ having a space group of Amma with the lattice parameters of *a* = 2.850, *b* = 0.3805, and *c* = 2.051 nm^19^. This is a ReO_3_-type structure and the metal–oxygen octahedra (MO_6_) form 3 × 4 blocks, or slabs, referred to as the “block structure”. They join neighboring blocks via sharing of octahedral edges, meaning oxygen reduction, and form a rectangular column along the short *b*-axis direction. The four column faces are called a “crystallographic shear (CS) plane”. One of the present authors successfully imaged the cation arrangements of the orthorhombic Ti_2_Nb_10_O_29_ crystal structure for the first time using HRTEM in the 1970s^10^. The research on the ternary Ti-Nb and Ti–W oxide has been resumed and extensively investigated as the candidate electrodes for Li batteries^1–8^.

The charge balance of a Ti_2_Nb_10_O_29_ crystal can be established as Ti^4+^ and Nb^5+^. The structural entity of the crystal is only MO_6_ octahedron. This implies that the charge states of the metal ions of the ternary oxide examined in this work will vary, particularly at the CS planes where reduction will take place; thus a cation ordering can be expected. In his XRD analysis of the crystal structure, Wadsley^19^ assumed that the Ti and Nb ions were evenly distributed among the octahedral sites because their charge states were unknown. However, Von Dreele and Cheetham^20^ examined the crystal utilizing an earlier Rietveld neutron powder diffraction which has an advantage over the X-ray diffraction method particularly for oxygen ion identification because of its much larger scattering factor than that of the X-ray. They concluded the Ti ions were located preferentially near the CS planes, while the Nb ions were located near the center of the ReO_3_-type blocks. More recently, a cation chemical ordering has been discussed for Nb-W oxide crystals using the neutron method via first-principle calculations^8,21^. However, the theory and the experiments were not entirely in agreement such as on cation occupancy ratios.

The spatial resolution of scanning transmission electron microscopy (STEM), as well as energy dispersive X-ray spectroscopy (EDS), has been greatly improved due to the introduction of a spherical aberration corrector and a new silicon drift detector (SDD) giving rise to high efficiency of X-ray detection. As a result, the elemental mapping of a crystal at an atomic resolution is now possible^22^. The first atomic-resolution elemental mapping of single atoms was demonstrated in STEM combined with electron energy loss spectroscopy (EELS)^23^ and imaging of atomic columns in a crystal^24^. The EELS method however is limited to ultra-thin films. The first atomic resolution STEM-EDS images were reported by Watanabe et al.^25^ and the usefulness was demonstrated in our previous publication, in which we have reported the cation ordering in Nb-W ternary oxide compounds with the tetragonal tungsten bronze (TTB)-type structure^26^. Although the quantification of the atomic resolution EDS-STEM imaging has not been established well because of fundamental problems with EDS emissions from a solid crystal such as electron channeling effect, we demonstrate here what we can do for atomic resolution EDS-STEM imaging in such a limited circumstance.

In the present work, we examined orthorhombic Ti_2_Nb_10_O_29_ crystals, utilizing the latest STEM in conjunction with EDS which was performed in the three principal crystal directions. In our new analyses of the cation ordering in Ti_2_Nb_10_O_29_ crystal using STEM-EDS, besides a similar result to that obtained by the earlier Rietveld powder neutron diffraction^20^, we also found some discrepancies in the detailed metal ion occupancies. In addition, we will present a new finding of displacements of the oxygen ions associated with multiple oxidation states of the cations at the CS planes, and also describe a diffuse scattering pattern in the electron diffraction (ED) pattern associated with the cation orderings.

## Results and discussions

### STEM-HAADF and STEM-EDS imaging in the [010] orientation

**Figure 1:**
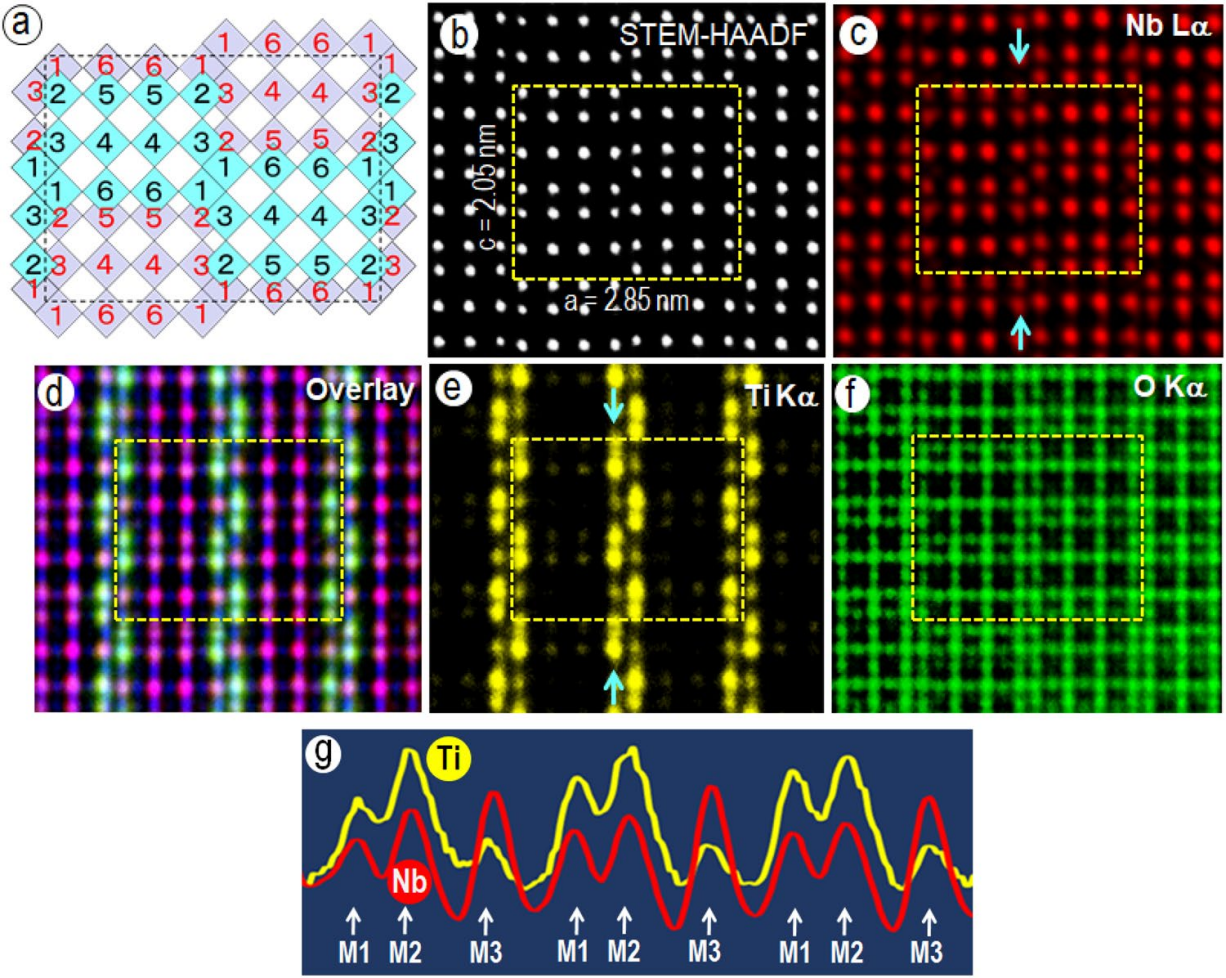
STEM-EDS images of an orthorhombic Ti_2_Nb_10_O_29_ crystal. (**a**) A model of the orthorhombic crystal viewed from the [010] direction. The numbers denote the metal atom sites. (**b**) A STEM-HAADF image of a Ti_2_Nb_10_O_29_ crystal in the same orientation as in (**a**), showing the metal atomic columns (white dots). (**c**) A STEM-EDS image recorded using the Nb Lα emission. (**d**) An overlay image of the three STEM-EDS images of Nb, Ti, and O, showing a segregation of the metal atomic columns in an ordered fashion. (**e**) STEM-EDS image recorded using the Ti Kα emission. (**f**) STEM-EDS image recorded using the O Kα emission. The images correspond to the exact same region of the crystal shown in (**b**). (**g**) The intensity line profiles, in an arbitrary scale, of Nb and Ti traced between the two cyan arrows drawn in the middle of (**c**) and (**e**).

The crystal structure of orthorhombic Ti_2_Nb_10_O_29_ projected in the [010] direction is illustrated schematically in Fig. 1a, in which two types of blocks of 3 × 4 MO_6_ (cyan and grey squares) are arranged in such a way that they overlap halfway with each other in the *b*-axis direction and the unit cell is outlined with a rectangle. The metal ion sites (M) are classified to six types labelled by the numbers 1–6, following Wadsley’s notations^19^, and the sites having the same number are crystallographically equivalent. In each octahedron, four oxygen ions are located at the corners of the square and the other two are located on the top and bottom at the center of the square (into and out of the plane of the page) and all octahedra form linear chains in the [010] direction.

A STEM image recorded from a thin region of a Ti_2_Nb_10_O_29_ crystal in the high-angle annular dark field (HAADF) imaging mode is depicted in Fig. 1b. The estimated specimen thickness was about 5 nm. The white dots correspond to the columns of metal–oxygen ions, –O–M– (denoted as M/O) parallel to the [010] direction and the positions of the dots match the metal ions in each octahedron in Fig. 1a. The oxygen–oxygen ion columns, –O–O– (denoted as O) are invisible in the HAADF mode. The STEM-EDS images shown in Figs. 1c, 1e, and 1f were recorded using Nb Lα, Ti Kα, and O Kα X-ray emissions, respectively, and Fig. 1d is their overlay image. The intensities of the original STEM-EDS elemental maps were measured in X-ray emission counts. The images were captured from the identical area of the crystal as shown in the HAADF image in Fig. 1b. The red dots in the Nb Lα image of Fig. 1c coincide exactly with the white dots in the HAADF image of Fig. 1b, implying that the Nb ions appear to be evenly distributed at all metal atom sites (columns). However, the intensities of the red dots revealed that some of them are slightly lower than the others. Similarly, the intensity variation in Ti distribution is more obvious. For easily comparing with the differences in intensity change in Nb and Ti distributions, the intensity line profiles of both Nb and Ti tracing along the row of the M/O columns between the two cyan arrows in Fig. 1c and Fig. 1e are drawn in the same figure (Fig. 1g) with an arbitrary scale. In Fig. 1g, red and yellow curves represent the intensity changes for Nb and Ti, respectively. It is noted that the highest peak in the Nb line appears at the M3 site but the peak at the same site in the Ti line becomes lowest among other Ti peaks.

Interestingly, the Ti ion containing columns appear in an ordered manner, as shown in Fig. 1e, although the columns contain both Nb and Ti ions. It was concluded that the Ti ions are present selectively at the octahedral sites denoted by octahedra 1 and 2 in Fig. 1a. These octahedra are located at the corners of the 3 × 4 blocks, where two CS planes intersect and octahedron 3 is shared with two neighboring octahedra 1 and 2 at their edges. It is noted, however, that the metal–oxygen octahedra 5 and 6 which connect to others via sharing only one edge, are occupied dominantly by Nb ions.

The results of measurements of the Ti occupancies performed by EDS are tabulated in Table 1 together with the data obtained by the Rietveld neutron powder diffraction study^20^. The quantification of atomic resolution EDS-STEM images has not been established because of some complexities of X-ray emission mechanisms in solids and therefore our measurements here are quantitative only for the relative concentrations of the same cations. In order to directly compare our result with that from the Rietveld neutron powder diffraction the Ti occupancies were recalculated according to the total metal ion occupancy ratios of Ti/(Ti + Nb) satisfying the composition of Ti_2_Nb_10_O_29_. It is noted that the relative occupancy values for M1-M6 sites are not affected after the recalculation. The two data sets, our EDS and the neutron diffraction, reveal some differences especially in the ratio of M1 and M2 site. The difference between site M2 and M1 was 24.5% using the neutron diffraction method, but only 5.5% using the EDS method. This discrepancy might be caused by the following two reasons: the chemical compositions of the specimens used in the two experiments were different or although they have same total Ti/Nb ratios, the local distributions of Ti/Nb differ due to the occasional intergrowth of a TiNb_2_O_7_ phase which was confirmed in our specimen. It should be mentioned here that the recently published paper pointed out a discrepancy between the neutron diffraction experiment and the DFT calculation on the similar complex Nb-W oxide of the block structures ^21^.

**Table 1:** Metal occupancies measured from the STEM-EDS images of a Ti_2_Nb_10_O_29_ crystal.

Ti occupations (%) from XEDS and neutron diffraction						
Methods	Metal					
	M1	M2	M3	M4	M5	M6
EDS	23.7	29.2	16.3	8.3	11.2	11.2
Neutron diff	15.5	40.0	12.7	4.5	10.7	16.8

Figure 1f depicts a STEM-EDS image acquired using the O Kα emission, and shows that the distribution of the oxygen ion columns corresponds to the oxygen ion sites located at the corners and apexes of each octahedron (see Fig. 1a). In the [010] orientation, there are two types of oxygen atomic columns, M/O and O. As both columns contain the same number of oxygen ions separated by almost the same distances, it is expected that they would have the same X-ray emission intensities. However, the oxygen column images are not uniform as seen in Fig. 1f. The similar intensity anomaly of the X-ray emission from the oxygen ions was also observed in the other orientations, [100] and [001].

### STEM-EDS imaging in the [100] and [001] orientations

**Figure 2:**
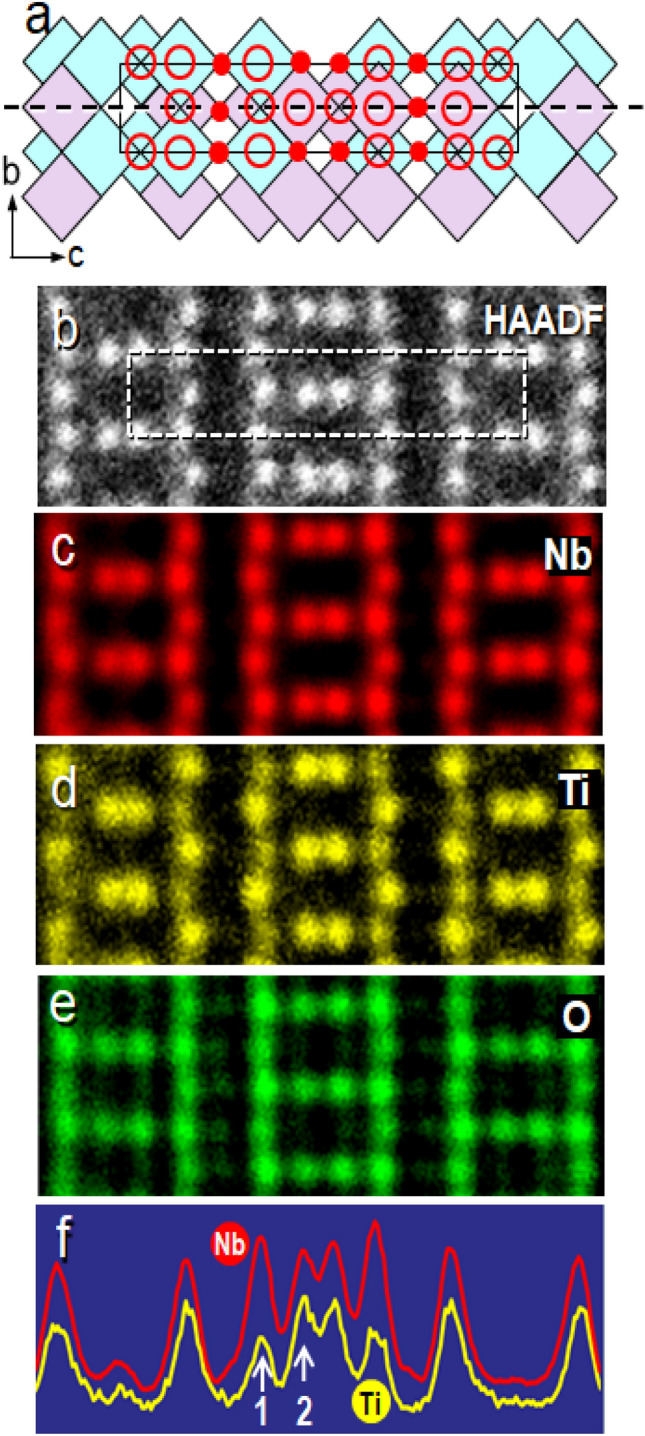
STEM-EDS images of an orthorhombic Ti_2_Nb_10_O_29_ crystal, acquired from the [100] direction. (**a**) A schematic model for an orthorhombic crystal viewed from the [100] direction, where the open and solid red circles denote metal/oxygen atomic columns and oxygen columns, respectively. (**b**) A STEM-HAADF image of the crystal from the [100] direction, showing only the metal atomic columns (large white dots). (**c**), (**d**), and (**e**) STEM-EDS images recorded using the Nb Lα line, Ti Kα line, and O Kα emissions, respectively, from the [100] direction. The images correspond to the same region of the crystal shown in (**b**). (**f**) Intensity line profiles of (**c**) and (**d**) scanned along the dotted line in (**a**).

Figure 2a illustrates a model for a crystal structure of Ti_2_Nb_10_O_29_ projected in the [100] direction. A STEM-HAADF image corresponding to the model is shown in Fig. 2b, in which the white dots match to the M/O columns, and no O columns are recognized. Figures 2c–e show STEM-EDS images recorded using Nb Lα, Ti Kα, and O Kα emissions, respectively. Two features should be mentioned here. First, the intensity profile of Nb distributions in Fig. 2c (the red curve in Fig. 2f, scanned along the dotted line in Fig. 2a), revealed two different heights of the peaks. This implies that the M/O columns contain two different amounts of the Nb ions as described below. Second, there are two distinct levels of the intensities of yellow dots representing Ti distributions in Fig. 2d, low and high. The lower intensity peaks correspond to the M/O columns containing both of the M3 and M4 sites and the higher intensity peaks correspond to the overlap of the M1 and M6, or the overlap of the M2 and M5. The highest peak positions indicated by the white arrow 1 in the red curve (Fig. 2f) is matched with the lowest peak in the yellow curve, suggesting that their corresponding M/O columns are dominated by Nb ions. The results are consistent with the observation on the [010] orientation (see Table 1). The metal ion ordering in a Ti_2_Nb_10_O_29_ crystal will be discussed in Sect. 2.4. The distribution of oxygen ions is presented in Fig. 2e. They are located at the M/O columns (red open circles in Fig. 2a) and the O columns (red solid circles in Fig. 2a). As was mentioned in the previous section, these two kinds of M/O and O columns seem to emit different amount of O Kα X-ray. The reason for the anomaly in the intensity of the oxygen Kα emission will be discussed below.

**Figure 3:**
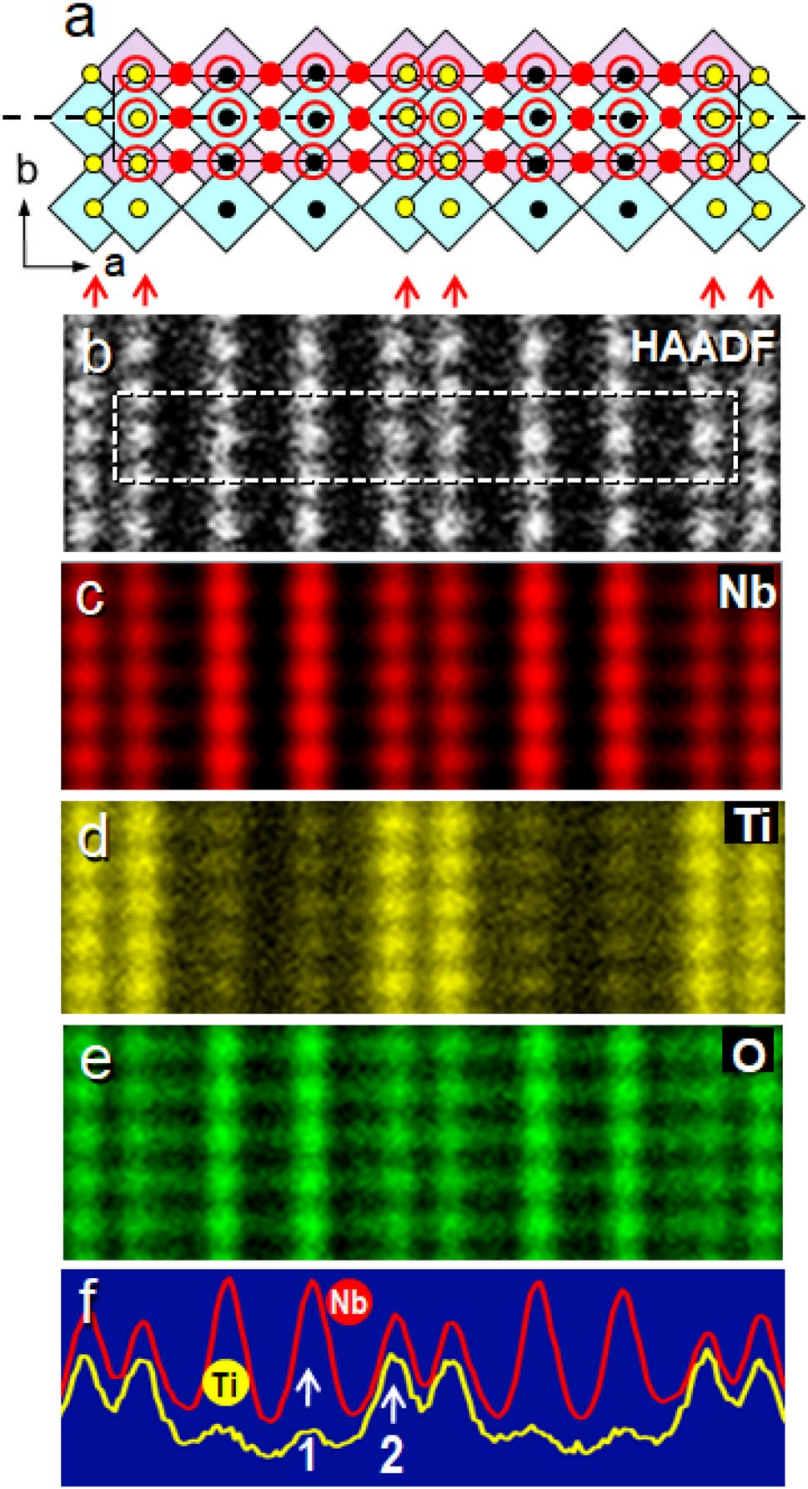
(**a**) A schematic model for an orthorhombic crystal viewed from the [001] direction. (**b**) A STEM-HAADF image of the crystal from the [001] direction. (**c**), (**d**), and (**e**) STEM-EDS images recorded using the Nb Lα, Ti Kα, and O Kα emissions, respectively, from the [001] direction. (**f**) Intensity line profiles of (**c**) and (**d**) scanned along the dotted line in (**a**).

STEM-EDS images of the atomic columns of Nb, Ti, and O ions in the [001] orientation are depicted in Figs. 3c–e. In this orientation, there are also two types of atomic columns of M/O and O ions as illustrated in Fig. 3a but the amount of oxygen ions in the O columns is a half of the O columns in the [010] and [001] orientations. The M/O columns are the bright dots in the STEM-HAADF image (Fig. 3b) and they match well those red dots in the Nb Lα EDS image (Fig. 3c). In Fig. 3c, the arrays corresponding to those indicated by the arrows in Fig. 3a are slightly fainter than the other arrays, implying that these columns contain less amount of Nb ions. The difference can be seen more explicitly in the Ti Kα EDS image shown in Fig. 3d. The intensities at the columns corresponding to those indicated by the arrows in Fig. 3a are much higher than those at the other columns, suggesting that the columns indicated by arrows contain more Ti ions than the other columns (see the white arrow 2 in Fig. 3f). These columns contain three kinds of the metal sites, M1, M2 and M3 which were populated more with Ti ions than the other M4, M5 and M6 (see Table 1). It should be noted that the octahedra indicated by arrows in Fig. 3a are joined by edge-sharing at two different edges on each octahedron.

The O Kα EDS image is depicted in Fig. 3e. The columns only contain oxygen atoms, O, (denoted by red solid circles in Fig. 3a) are quite faint, while those of the M/O columns (open circles) are clearly visible. The difference in the contrast is due to the fact that the O columns contain only a half of the numbers of oxygen ions in the M/O columns in the [001] orientation. However, an intensity anomaly in the oxygen Kα emission was found at the M/O columns with different Nb/Ti ratios, specifically, the Nb rich M/O (with higher Nb/Ti ratio) and the obviously seen Ti containing M/O columns (with lower Nb/Ti ratio) denoted by red arrows in Fig. 3a. In Fig. 3e, the former is brighter than the latter, suggesting that the oxygen ions in the Nb-rich columns appear to emit more X-rays. One possible reason for the anomaly could be associated with the electron channeling effect for the incident electron probe. The columns containing heavier Nb ions attract more probe electrons than the other columns containing relatively more lighter Ti ions, and therefore the probe electrons appear to be enhanced in the Nb rich columns. Here it should be reminded that both columns contain the same amount of oxygen ions. The quantitative arguments on the EDS measurements are beyond the scope of the present report and will be reported in another occasion.

### STEM-ABF imaging of oxygen columns in the [010] orientation

**Figure 4:**
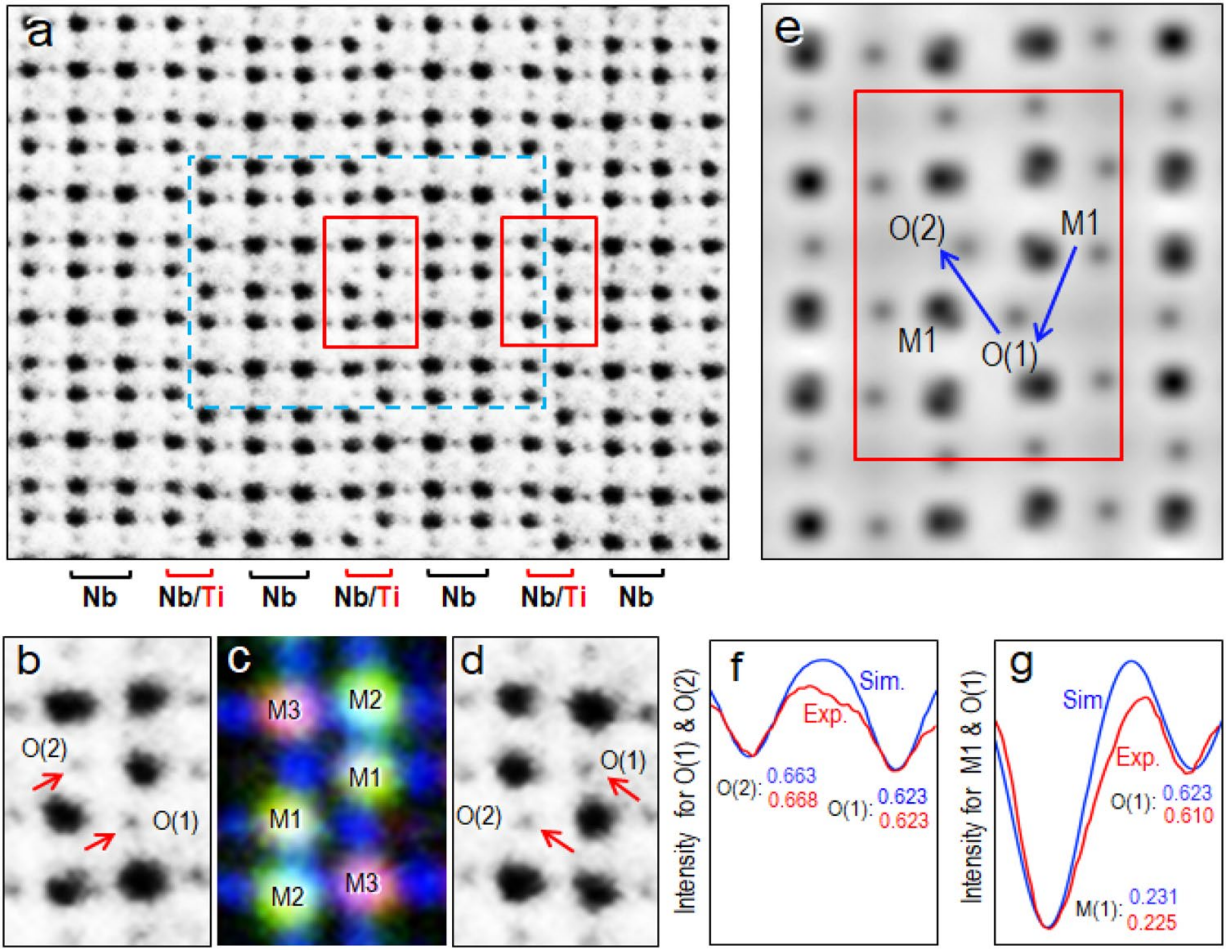
STEM-ABF image of an orthorhombic Ti_2_Nb_10_O_29_ crystal viewed from the [010] direction. The unitcell was depicted by the dashed rectangle. (**a**) A STEM-ABF image showing metal–oxygen ion columns (Black and large dots) and oxygen ion ones (faint grey small dots around the black dots). (**b**) An enlarged STEM-ABF image taken from the center of the unitcell (enclosed by a red rectangle). (**c**) An overlay image of the three STEM-EDS images of Nb, Ti, and O, corresponding to the one shown in panel (**b**), which includes M1, M2 and M3 metal–oxygen columns as well as the oxygen columns (blue dots). (**d**) An enlarged STEM-ABF image near the edge of the unitcell enclosed by a red rectangle (right) in (**a**) showing c-glide symmetry image of (**b**). (**e**) A simulated image of [010] direction with O(1), O(2) and M(1) indication. (**f**) A comparison of the image intensities from the O(2) and O(1) columns between the simulated (blue line labeled with Sim.) and experimental (red line labeled with Exp.) images. The averaged intensities of O(2) and O(1) from both observed (red numbers) and simulated (blue numbers) images are also inset. (**g**) A comparison of the M(1) and O(1) columns between the simulated (blue line labeled with Sim.) and experimental (red line labeled with Exp.) images with the averaged intensities (red numbers: experimental and blue numbers: calculated from the simulated image) inset. The commercially available software “Tempas (ver. 3.0.42)” (https://www.totalresolution.com/index.html) was used for the simulation of the STEM-ABF image (Fig. 4e).

We mentioned that there are two types of oxygen columns in the [010] orientation and the STEM-HAADF imaging mode is not capable for detecting oxygen columns. This weakness is compensated by STEM-ABF imaging mode that is suitable for light elements such as oxygen ions columns. Figure 4a depicts a STEM-ABF image of the Ti_2_Nb_10_O_29_ crystal oriented in the [010] direction. The black dots represent the M/O columns and the faint small grey dots between the black dots are corresponding to the oxygen columns. Some of their enlarged images are shown in Fig. 4b and 4d, which were taken from near the center indicated by the two red rectangles, respectively, in Fig. 4a. The large black blobs are identified as M1, M2 and M3 metal sites and their corresponding STEM-EDS overlay image (Fig. 1d) is shown in Fig. 4c.

Here let’s look into the details of the pair of the faint grey dots indicated by the red arrows in Figs.4b and d, which correspond to the O(1) and O(2) columns. It was found that the contrast of the O(1) is slightly darker than that of the O(2). The difference in the contrasts was recognized in all the crystallographically equivalent O(1) and O(2) columns. One of such examples was depicted in Fig. 4d, which was taken from the right side region indicated by the red rectangle in Fig. 4a. The image has c-glide symmetry to the one shown in Fig. 4b.

The difference in the two image intensities is explicitly demonstrated in the line profile tracing across two peaks, the O(1) and O(2), as shown by the red curve in Fig. 4f. In order to quantitatively show the intensity differences between O(1) and O(2) the STEM-ABF image shown in Fig. 4 is normalized having intensity values from 0 to 1, the darker the smaller value. The averaged intensity values measured over 30 sets of the two peaks are 0.623 for the O(1) and 0.668 for the O(2), and the difference is 4.5%. To perform a cross check, we also measured the intensities of the M(1) columns and at the mean while the intensities of the O(1) in the most nearby as shown by the red arrows in Fig. 4b and 4d. The averaged intensity values of the M(1) and O(1) columns are 0.225 and 0.610, respectively.

In order to elucidate the origin of the contrast differences in O columns, we compared the experimental images with the simulated STEM-ABF images. We assumed that the image intensities of different O columns are proportional roughly to a static potential field of the atom when they are observed under the same condition (such as the same thickness and defocus, etc.). Under this assumption, there will be two possibilities causing the difference in the image contrasts, the presence of oxygen atom vacancies and displacements of the atoms from the center of the oxygen columns. Here we focus on the displacements because the oxygen vacancies might not exist in our sample having a stoichiometric composition. If all oxygen atoms stay at the center of the oxygen column without any displacement, the image of the oxygen column will show a maximum contrast. This is the case for O(1) column, however, in the O(2), some of the oxygen atoms might have been displaced from the center of the column. The displacements mean that the potential field of the displaced oxygen atoms in the column will be spread out around the column center and therefore the image intensity of the column decreases.

In the STEM-ABF image simulation of the Ti_2_Nb_10_O_29_ crystal, Debye–Waller (D-W) factors were introduced in the displacement of the oxygen atoms in O column to vary displacement amplitudes of the oxygen atoms. We used the measured occupancy rate for the M1 column (see Table 1) in the STEM-ABF image simulation. One STEM-ABF simulated image that was performed by using the actual observation condition is shown in Fig. 4e. The simulated STEM-ABF image is also normalized to have intensities value between 0 and 1 with the brightest intensity 0 and darkest intensity 1. The intensity profiles tracing the O(1) and O(2) , and also M1 and O(1) were measured along the blue arrows. In Fig. 4e, the intensity for measured from the M1 column was 0.231 which is close to the experimental value of 0.225, and meanwhile the intensity for O (1) column was 0.623, which is also close to the experimental value of 0.610. These intensity profiles from simulated and observed images are shown by the blue and red curves, respectively, in Fig. 4f and 4g. After confirming the agreement between the experiment and calculation, we adopted the D-W factor for the oxygen ion in the O(1) column as a standard and sorted a D-W factor for the oxygen ions in the O(2) column to be matched with that of the experimental value 0.668. We found that a good agreement was obtained when the D-W factors for the oxygen ions in the O(1) and the O(2) columns were 0.5Å^2^ and 2.5Å^2^, respectively as shown in Fig. 4f. The reason for the larger displacement of the oxygen atoms in the O(2) columns could be associated with the ordering of Ti and Nb ions in the M1 columns in the [010] direction, which will be discussed in the next section.

### b-axis modulation due to cation ordering

**Figure 5:**
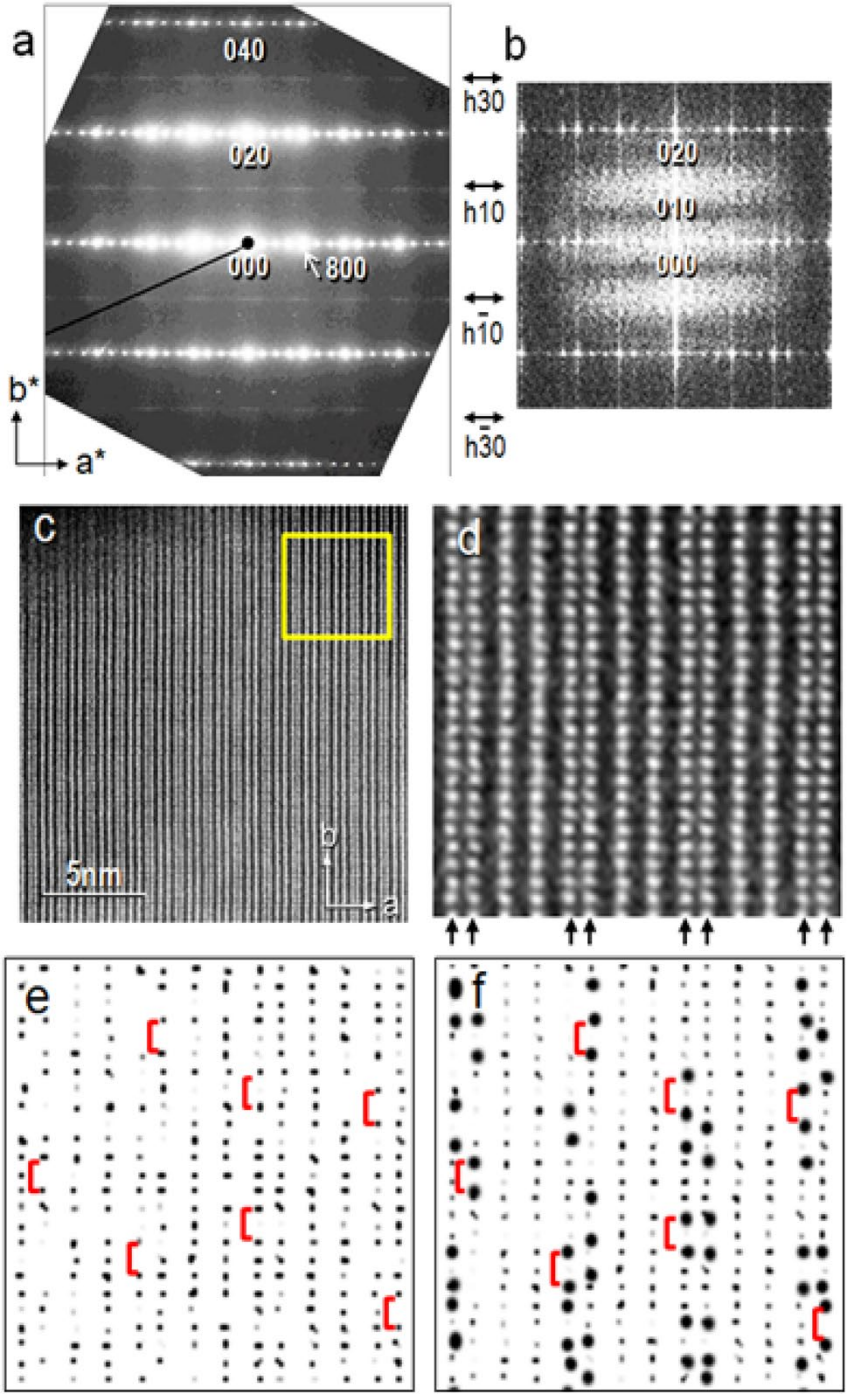
(**a**) An electron diffraction pattern recorded from a Ti_2_Nb_10_O_29_ crystal oriented in the [001] direction. Note the diffuse lines appearing at reciprocal points (0,2n + 1,0), which are forbidden reflections for the space group Amma of this crystal. (**b**) A Fourier-transformed image of a processed STEM-HAADF image reproduces a similar but fuzzy diffuse pattern to (**a**). (**c**) A STEM-HAADF image acquired from a Ti_2_Nb_10_O_29_ oriented in the [001] direction. (**d**) An enlarged image of the region squared in (**c**). The arrow heads indicate the M/O column arrays containing more Ti ions. (**e**) A reversed and enhanced contrast image of the panel in (**d**). (**f**) The paired dots are highlighted by adding large black dots on them. Some of the pairs are indicated by red brackets in (**e**) and (**f**).

The preferential presence of Ti ions in the octahedra located at all four corners of the 3 × 4 blocks (see the M1 and M2 sites in Fig. 1a) on the CS planes was confirmed experimentally as mentioned in the previous sections. Here, we consider a three-dimensional distribution of Ti ions. Figure 5a shows an electron diffraction (ED) pattern recorded from a Ti_2_Nb_10_O_29_ crystal oriented in the same [001] direction as the STEM-HAADF image shown in Fig. 3b. The sharp spots were indexed as (h, k, 0) reflections with h, k = 2n (n: integer) for the space group Amma of this crystal. The pattern revealed weak diffused lines at (h, 2 k + 1, 0) as indicated by arrows. These reflections are forbidden for the crystal space group. Similar diffuse scattering lines have been observed in a Nb_12_O_29_ crystal with excess oxygen that is isomorphous with the orthorhombic Ti_2_Nb_10_O_29_ (see Fig. 2d in the study by Iijima (16)). In Nb_12_O_29_ crystal, the origin of the diffuse scattering was explained in terms of a formation of defective tetrahedral sites for Nb ions which occurred near the corners of the 3 × 4 blocks and at every unitcell along the *b*-axis. The occurrence of the defects breaks the *b*-glide symmetry of the Amma crystal symmetry and therefore, the (h, 2 k + 1, 0) reflections can be allowed. Although such defective tetrahedral sites were not found in this study, the origin of the diffuse scattering would be analogous and associated with the intensity anomaly of the O(2) columns mentioned in the previous section, To understand the origin of the diffuse lines in the ED pattern, we performed a Fourier-transform (FT) of the STEM-HAADF images shown in Fig. 5c, a wider view of Fig. 3b. However, the FT image failed to reproduce the diffuse lines presumably because the SN ratio is not high enough. Next, we processed the original images as follows: the image contrast was reversed and further enhanced. As an example, the enlarged image of the area shown by the square in the Fig. 5c was depicted in Fig. 5d, in which the arrays of the M/O columns containing more Ti ions are indicated by the pairs of the arrows at the bottom. Subsequently its contrast was reversed and enhanced (Fig. 5e). Here we concern with the arrays of the dark dots, corresponding to the Nb-Ti columns parallel to the *c*-axis (vertical to the page and denoted as (Nb-Ti)_001_), indicated by the yellow solid circles and the arrows in Fig. 3a. The processed image revealed that the image intensities of the dots in each array fluctuated and some of the dots almost disappeared. The contrast fluctuation would be associated with different metal occupancy ratios of the (Nb-Ti)_001_ columns and the darker dots mean less Ti ions and more Nb ones (richer Nb-Ti)_001_. We found that the darker dots appeared frequently in pair, and they are separated by the length of the *b* axis. Those pairs were highlighted further by drawing large black dots on them, as depicted in Fig. 5f, in which some of the pairs were marked by the red brackets. The pairing means that the (Nb-Ti)_001_ columns are sandwiched between the (richer Nb-Ti) _001_ columns. The image processing was sampling area of the STEM-HAADF image used for the FT is not large enough to applied manually to the whole image of Fig. 5c, and its FT pattern was examined. The result is shown in Fig. 5b, which seems to reproduce the diffuse pattern at (h, 2 k + 1, 0) similar to the one shown in Fig. 5a. The pattern is not sharp lines as for the electron diffraction pattern. This might be due to the fact that the produce the sharp diffuse lines unlike electron diffraction experiments ^27^. It can be said that the diffused patterns originated from the occurrence of the pairing dots, or a correlation among the cations within the (Nb-Ti)_001_/O columns. Now we concluded that the periodicity of a half of the *b*-axis was locally doubled, and therefore the Amma crystal symmetry was lost, suggesting that the (Nb-Ti)_001_/O columns may occur occasionally in pair throughout the crystal.

### A proposed model for the cation ordering

**Figure 6:**
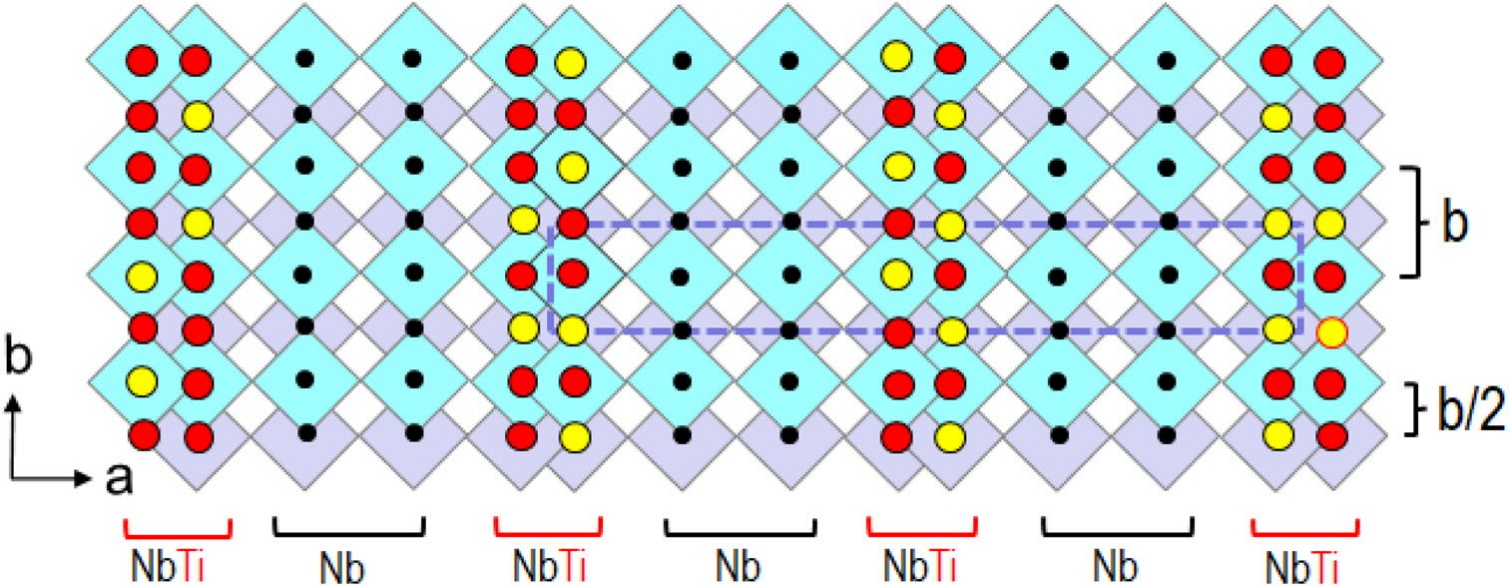
A schematic model for the cation ordering in the [001] direction of the orthorhombic Ti_2_Nb_10_O_29_ crystal. The cross-sectional view from the [001] direction. The three types of the M/O octahedra columns are indicated by black dots (Nb rich), red balls (Nb and Ti, denoted as Nb + Ti), and yellow ones (Nb and relatively richer Ti than those in red balls, named Nb + Ti’), drawn at the center of each octahedron. The yellow balls tend to be sandwiched with red ones, implying a short-range-order.

Taking into account all experimental results as described above, in addition to the ordering of cations on the *a*-*b* plane, we proposed a model for the cation ordering in the three dimensions as illustrated in Fig. 6. There are three types of M/O columns present in the [001] direction (vertical to the page), which contain a different amount of Ti ions The first types is Nb richest columns which are distributed evenly throughout the columns (denoted as Nb rich, small black dots). The second one is Nb ion columns containing a slight amount of Ti ions (denoted as Nb + Ti, indicated by red balls). The third one contains relatively more Ti ions than those in Nb rich and Nb + Ti columns and is denoted as Nb + Ti’ (yellow balls). Nb + Ti and Nb + Ti’ columns tend to form in a partially ordered manner, that is, the Nb + Ti’ columns are sandwiched between the Nb + Ti columns. The perfectly ordered structure is a long-range-ordering for which the yellow balls (the Nb + Ti’ columns) and red ones (Nb + Ti columns) appear alternately along the [010] direction. However, in the present case the ordering is not perfect but partial, or a short range in the [010] direction.

The ordering might be associated with the displacements of the oxygen ions in the O(2) columns described in the  "b‑axis modulation due to cation ordering" section. These oxygen ions are situated in Nb-Ti oxygen columns at the corner of the 3 × 4 blocks and bonded to the M1 ions. The local arrangement of the two types of the cations Ti + 4 and Nb + 5 might be resulted in redistribution of the charges including those of the oxygen ions in the O(2) columns. The cation orderings should be determined by electronegativity, chemical bond and atomic numbers of the cations. The local modification of the M1 and M3 cation sites might not be so drastic as a formation of the tetrahedral sites that were found in the oxygen excess Nb_12_O_29_ crystals^12,16^.

## Conclusions

The latest atomic resolution STEM-EDS method was successfully utilized to solve an order–disorder problem with Ti-Nb-O complex metal oxide crystals. The method has been proved to provide useful information in crystallography complimenting the conventional X-ray or neutron diffraction methods particularly in disordered crystals and powder crystals.

It was experimentally proved that Ti and Nb ions in a ternary oxide of Ti_2_Nb_10_O_29_ segregate in an ordered fashion, and Ti ions tend to aggregate at the octahedral sites located at the intersections of two CS planes and in this way, the mixing of Ti and Nb of multivalent ions would stabilize the crystal structure of Ti_2_Nb_10_O_29_.

The ordering of Ti ions determined by the present study generally supports the results reported in the neutron diffraction study although some of the metal occupancy ratios differed. This might be due to the fact that the conventional powder diffraction methods are not entirely adequate especially for powder crystals containing occasional intergrowth of TiNb_7_O_10_ and for crystals with a large unitcell (Ti_2_Nb_10_O_29_, *a* = 2.850, *b* = 0.3805, and *c* = 2.051 nm).

Beside an ordering of Ti in *b* plane, a new finding is the partial ordering of Ti ions in the *b*-axis direction at the intersecting CS planes on the *a* and *c* planes. The ordering was not a long range but short-range in such a way that the Ti/O octahedron and Nb/O ones repel each other in adjacent M/O columns along the [010] direction.

## Method

The investigated Ti_2_Nb_10_O_29_ sample was composed of yellowish-white powder crystals that prepared by Allpress 50 years ago^28^, that had been studied by one of the present authors^10^. The crystal is isostructural with Nb_12_O_29_ crystal with a slight excess of oxygen which showing a dark grey color.

For the TEM observation, the powder samples (less than 1 μm in size) were placed onto a standard TEM holey carbon-coated support grid and a thin crystallite was located and aligned with the observational directions of one of the three principle crystal axes. All electron microscope images presented here were recorded at STEM mode using JEM-ARM300F (JEOL Co.) equipped with dual silicon drift detectors (SDDs) having a total detection area of 2 × 158 mm^2^, operated at 300 kV. All STEM-HAADF and ABF images were taken under the following conditions: The estimated probe current and probe sizes were 10–20 pA and < 0.1 nm in diameter, respectively. The original image sizes were 1 k × 1 k pixels. The images were raw data, which were not processed with any filters. All EDS maps were recorded under the following conditions: The estimated probe current and probe sizes were the same as the STEM-HAADF and ABF images. The mapping size was 256 × 256 pixels, and the dwell time was 10 μs/pixels. The measurement time of each mapping was 45 to 60 min, depending on the field of view. The EDS elemental images were processed with the Wiener filter to enhance the contrast. The intensities of STEM-ABF images were normalized during the calculations of intensity profiles of M(1), O(1) and O(2).

The commercially available software “Tempas (ver. 3.0.42)” (https://www.totalresolution.com/index.html) was used for the simulation of the STEM-ABF image (Fig. 4e).
